# 63 Left out in the Cold: The Impact of Psychosocial Comorbidities on Victims of Frostbite

**DOI:** 10.1093/jbcr/irac012.066

**Published:** 2022-03-23

**Authors:** Aurelie Tran, Hannah Glick, Mary R Shen, Amanda Bettencourt, Gary Vercruysse

**Affiliations:** University of Michigan Medical School, Ann Arbor, Michigan; University of Michigan, Ann Arbor, Michigan; University of Michigan, Ann Arbor, Michigan; University of Pennsylvania School of Nursing, Philadelphia, Pennsylvania; University of Michigan, Ann Arbor, Michigan

## Abstract

**Introduction:**

Patients with psychiatric or substance use disorders (SUD) and those experiencing homelessness have been identified as populations likely disproportionately affected by frostbite injury. However, the literature is sparse in regards to morbidity and mortality in these patients. As such, we sought to examine and characterize factors associated with worse outcomes and increased resource utilization in this patient population.

**Methods:**

Adult patients admitted to a single ABA-accredited burn center for frostbite between 2013-2021 were identified using an institution-specific data registry. A retrospective chart review was conducted on patients meeting inclusion criteria, as identified by ICD-10 and ICD-9 codes. The primary outcome was morbidity and mortality associated with frostbite, including hospital length of stay, number of operations, and readmission. Chi-square and t-tests were utilized to compare patients with and without SUD (alcohol, drug, or positive urine drug screen), psychiatric disorders, or homelessness.

**Results:**

In total, 54 patients were identified (70% male), 19% had documented non-alcoholic SUD, 50% had alcohol use disorder, and 14% were homeless. No significant differences were found between these patients and others in terms of the number of operations or amputations required. However, patients with positive SUD screen (32.0% vs 8.0% p=0.03), positive UDS (46.7% vs. 0%, p=0.015), psychiatric disorders (27% vs. 0%, p=0.034), active drug use (50% vs 14.3%, p=0.01), or homelessness (50% vs 15%, p=0.026) were more likely to be readmitted with wound infections or progression of gangrene. Finally, patients with psychiatric disorders were more likely to require additional operations (1.8 vs 0.6, p=0.02) and longer length of hospital stay (16.0 +/- 2.9 vs 7.7 +/- 2.8, p=0.046).

**Conclusions:**

Our results suggest significant differences in resource utilization and morbidity between those with and without a history of SUD, psychiatric disorder, or homelessness. Subsequent allocation of resources should target outpatient needs of at-risk patients to avoid similar outcomes. Future research should be focused on elucidating reasons for these differences which may include issues accessing follow-up care, inability to adhere to wound care, and more.

